# Fad diets, schizophrenia, and politics: An internet frenzy that highlights broader concerns

**DOI:** 10.1371/journal.pmen.0000619

**Published:** 2026-06-03

**Authors:** Taylor Locke

**Affiliations:** Schizophrenia Comprehensive Care Initiative, National Alliance on Mental Illness (NAMI) New York State, Albany, New York, United States of America; PLOS: Public Library of Science, UNITED KINGDOM OF GREAT BRITAIN AND NORTHERN IRELAND

There has been much online discourse over claims made in February of this year that schizophrenia can be cured using keto diets [1]. Lost in the noise of these claims was something far more important: the reality of schizophrenia as a complex medical condition and the responsibility we all share when discussing serious illness in politically charged spaces.

While nutrition is an important and evolving area of mental health research, there is currently no established cure for schizophrenia via ketogenic diets, or by any other known methods [2]. This viral claim reveals broader concerns about how medical information spreads online – and how quickly nuance disappears when health, politics, and social media collide.

## The Keto diet: From legitimate therapy to modern fad

The ketogenic diet isn’t new. It originated in the 1920s as a legitimate medical therapy for epilepsy and remains an evidence-based treatment for certain seizure disorders today [3]. In controlled medical settings, it can be highly effective.

In recent years, however, keto has evolved into something else: a popular fad diet. It is widely promoted for weight loss, metabolic optimization, and a growing list of other health claims. Yet the diet is highly restrictive and difficult to maintain without professional supervision. Long-term adherence can raise concerns related to lipid levels, nutrient deficiencies, and overall cardiovascular health [4].

This is especially relevant when discussing schizophrenia, where individuals are already at significantly increased cardiovascular risk [5]. Additionally, the keto diet was developed as an adjunct to medication or for drug-resistant cases – not as a replacement for standard treatment. Encouraging people with serious mental illness to stop prescribed medication in favor of a dietary trend is unsupported by evidence and potentially dangerous, particularly given the risks of adverse effects when abruptly discontinuing antipsychotics.

Research in metabolic psychiatry is ongoing, but current evidence is preliminary. Small pilot studies do not establish efficacy, cure, or long-term safety. Until stronger data is available, ketogenic interventions should be pursued only with careful medical supervision, and any medication changes should always be made in consultation with a qualified healthcare professional.

## Understanding schizophrenia

Schizophrenia is a chronic brain disorder with multifactorial origins. While the exact causes are unknown, genetic predisposition, environmental stressors, and neurobiological changes have all been shown to contribute [6]. Symptoms are typically grouped into three domains:

- Positive symptoms such as hallucinations and delusions
- Negative symptoms such as diminished motivation or social withdrawal
- Cognitive symptoms affecting memory, attention, and executive function

There is currently no known cure. However, that does not mean there is no hope.

Standard treatments – including antipsychotic medications, psychotherapy, and community support – help many individuals achieve stability and meaningful improvement. Recovery does not necessarily mean the complete absence of symptoms. More often, it means learning to manage them, prevent relapse, and build a fulfilling life despite challenges.

Nutrition and physical health absolutely matter. The relationship between metabolism and mental health is a legitimate area of research. There are also complementary and alternative health approaches that may support symptom management when used alongside standard care. Some dietary strategies – particularly omega-3 fatty acid supplementation – have a stronger research base than many trending interventions and may offer modest benefits for certain individuals [7].

However, promising hypotheses are not the same as proven treatments. Improvement in some symptoms does not equal elimination of disease. And anecdotal success stories do not replace large-scale, peer-reviewed evidence.

## When “Cures” go viral

Health claims spread fastest when wrapped in political or ideological narratives. In polarized spaces, certainty replaces nuance, and complex psychiatric conditions are reduced to simple solutions. Nowhere is this more concerning than in discussions of chronic mental illness, where the language of “cure” implies finality in a field defined by biological complexity and individualized care.

Influencers can amplify these risks. For example, prominent psychosis content creator Lauren Kennedy West has been heavily criticized by members of the schizophrenia community for allegedly encouraging followers to abandon psychiatric medications in favor of approaches like the keto diet [8]. Similarly, other influencers have faced scrutiny for rebranding medical misinformation and racist/ableist ideologies as “New Age” spirituality, framing these ideas as forms of self- enlightenment [9]. This rhetoric can be especially harmful to individuals who experience psychosis, as spiritualized conspiracy narratives may reinforce delusional thinking or paranoia.

The broader lesson here is one of media literacy. Before sharing or acting on bold medical claims, we should ask:

- Is this supported by peer-reviewed research?
- Are major medical organizations endorsing it?
- Is the language cautious and evidence-based, or absolute and sensational?

Public figures bear responsibility when discussing health. Media outlets must provide context. And individuals should consult qualified professionals before making any changes to their treatment. Medical decisions – especially those involving serious mental illness – should never be driven by viral internet claims.

## Beyond the headlines: Lives behind the diagnosis

Amid debates about diets and politics, it’s easy to forget the 23 million people living with schizophrenia worldwide [10]. They are students, parents, co-workers, and neighbors who pursue careers, build relationships, and contribute to their communities.

Recovery isn’t a miracle transformation but a gradual, ongoing process involving treatment, support, and accepting that sometimes it’s okay to not be okay. But despite the symptoms, stigma, and significant barriers, it is possible.

Unfortunately, harmful stereotypes persist, and it’s important to challenge them.

First and foremost, schizophrenia is not caused by poor parenting or personal weakness, nor are people who report symptoms just “doing it for attention.” Decades of research show that schizophrenia is a complex brain disorder shaped by genetic and environmental factors, with numerous brain imaging studies revealing distinct neurological patterns in those diagnosed [11–13].

There are also many misconceptions surrounding antipsychotics, including that they are addictive or that side effects are always worse than the illness. Antipsychotics are not generally considered addictive like common substances of misuse, though stopping them abruptly can cause withdrawal symptoms and increase relapse risk [14]. Side effects vary widely by medication and person. For many people, benefits outweigh harms, and as with any treatment plan, decisions should be individualized through shared decision-making.

Perhaps most damaging of all, is the belief that people with schizophrenia cannot live happy, healthy, and fulfilling lives. Stability, purpose, and joy are attainable with appropriate treatment and support. What individuals living with schizophrenia need is not viral cure narratives, but access to evidence-based care, community resources, and public discourse based on compassion rather than sensationalism.

## Looking forward – A call for responsible communication

Nutritional psychiatry is a legitimate and evolving field, and continued research into metabolism and mental health is worthwhile. However, claims of cures require rigorous, replicated evidence before being treated as fact. This moment is an opportunity to slow down, examine the evidence carefully, and recommit to responsible conversations about serious mental illness.

For readers seeking reliable information, organizations such as the National Alliance on Mental Illness (NAMI) in the United States model what responsible communication looks like in practice. NAMI provides accessible, evidence-based resources that are regularly reviewed, grounded in current research, and shaped by both clinical expertise and lived experience. Its schizophrenia information page, for example, presents symptoms, treatment options, and recovery in clear, non-sensational terms while emphasizing the importance of professional care and individualized decision-making [15].

Organizations and individuals must prioritize accuracy over virality, acknowledge uncertainty, cite credible evidence, and remain accountable for the impact of their words. Above all, medical decisions should always be made with qualified healthcare professionals. Conversations about schizophrenia must center dignity, science, and lived experience – not political amplification or online frenzy.

People living with schizophrenia deserve accuracy, compassion, and discussions grounded in careful evidence and human understanding.
